# Genome-Wide Association Studies Using Haplotypes and Individual SNPs in Simmental Cattle

**DOI:** 10.1371/journal.pone.0109330

**Published:** 2014-10-20

**Authors:** Yang Wu, Huizhong Fan, Yanhui Wang, Lupei Zhang, Xue Gao, Yan Chen, Junya Li, HongYan Ren, Huijiang Gao

**Affiliations:** 1 Institute of Animal Science, Chinese Academy of Agricultural Science, Beijing, China; 2 Department of life sciences, National Natural Science Foundation of China, Beijing, China; Indiana University Bloomington, United States of America

## Abstract

Recent advances in high-throughput genotyping technologies have provided the opportunity to map genes using associations between complex traits and markers. Genome-wide association studies (GWAS) based on either a single marker or haplotype have identified genetic variants and underlying genetic mechanisms of quantitative traits. Prompted by the achievements of studies examining economic traits in cattle and to verify the consistency of these two methods using real data, the current study was conducted to construct the haplotype structure in the bovine genome and to detect relevant genes genuinely affecting a carcass trait and a meat quality trait. Using the Illumina BovineHD BeadChip, 942 young bulls with genotyping data were introduced as a reference population to identify the genes in the beef cattle genome significantly associated with foreshank weight and triglyceride levels. In total, 92,553 haplotype blocks were detected in the genome. The regions of high linkage disequilibrium extended up to approximately 200 kb, and the size of haplotype blocks ranged from 22 bp to 199,266 bp. Additionally, the individual SNP analysis and the haplotype-based analysis detected similar regions and common SNPs for these two representative traits. A total of 12 and 7 SNPs in the bovine genome were significantly associated with foreshank weight and triglyceride levels, respectively. By comparison, 4 and 5 haplotype blocks containing the majority of significant SNPs were strongly associated with foreshank weight and triglyceride levels, respectively. In addition, 36 SNPs with high linkage disequilibrium were detected in the GNAQ gene, a potential hotspot that may play a crucial role for regulating carcass trait components.

## Introduction

Single nucleotide polymorphisms (SNPs) are the genetic variant most commonly used in association studies. Successful attempts using genome-wide association studies (GWAS) to examine human diseases [1], especially for those studies using a case-control design, have made GWAS based on a single marker a widely accepted approach for gene detection in general. Inspired by this, subsequent large GWAS have been conducted focused mainly on complex traits, such as genetic defects and disease resistance or susceptibility [2]. These studies not only expanded applications of genome-wide molecular markers to marker-assisted selection but also provided important information for elaboration of the genetic mechanisms of these traits. Recent GWAS have explored economically important traits and breed characteristics of major livestock species [3], [4]. Thus, a wide range of successful applications of GWAS to animal breeding and genetics has been reported and many genes or markers affecting economic traits in animals have been identified. In beef cattle, for instance Japanese black cattle [5], Korean Hanwoo cattle [6], Korean beef cattle [7], and Australian taurine and indicine cattle [8], GWAS detected genetic variations associated with carcass and meat quantitative traits. Many significant main effects of SNPs were identified via simple linear regression and stepwise regression procedures. With more advanced genome sequencing and high-throughput SNP genotyping technologies, GWAS with individual markers will more efficiently and reliably determine underlying genetic mechanisms.

As specific sets of alleles observed on a single chromosome or part of a chromosome, haplotypes are inherited together with little chance of contemporary recombination. Numerous inherent merits have made haplotypes an integral part of genetic variants and available as super alleles. Recently, haplotypes have been identified that confer high susceptibility for schizophrenia [9], nicotine dependence [10], macular degeneration [11], and recurrent laryngeal neuropathy in horses [12]. Moreover, some studies [13], [14] assert that the analysis of haplotypes with the grouping and interaction of several variants is superior to any individual SNP analysis technique. Indeed, compared with individual SNP-based association studies, the use of multi-allelic haplotypes has significantly improved the power and robustness of association studies [15]–[17].

However, methodologies are always accompanied by drawbacks. For single marker analysis, only a small fraction of the genetic variation in quantitative traits can be explained using significant SNPs. One reason for this limitation is that the effects of individual SNPs are too small to pass the stringent significance criterion. Another reason is incomplete linkage disequilibrium (LD) between the genotyped SNPs and casual variants [18]. Haplotype-based GWAS are often hampered by the prohibitive time and costs required for haplotype inference [19]. Additionally, haplotype block structure and phase are rarely observed in genotyping data and may be subject to errors when inferred using statistical methods [20]. Moreover, when a block of genome contains a large number of haplotypes, the increased degrees of freedom within the block of the genome can erode statistical power [19].

Although haplotype association analysis has been conducted for many years using the human genome [21], little is known about the performance of this type of analysis in livestock. Indeed, the haplotype block structure and its distribution in the genome of cattle, especially studies based on high density SNPs, have been rarely reported [22]. Thus, the current study was performed to construct the haplotype structure in the bovine genome and to detect the relevant genes genuinely affecting a carcass trait component and meat quality trait.

## Results

### Haplotype blocks

**Figure 1:**
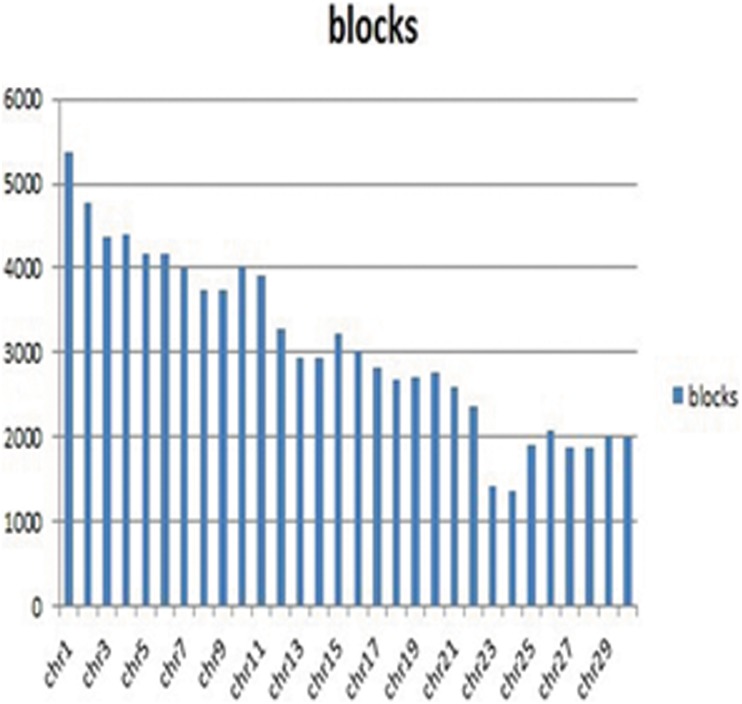
Number of haplotype blocks per chromosome.

We found that the whole cattle genome was partitioned into 92,553 haplotype blocks. The largest haplotype block consisted of 116 SNPs, the smallest block contained only 2 SNPs, and the average size of a block was 5.69. These haplotype blocks covered a total of 526,822 SNPs from the high density chips. The regions of high LD extended to approximately 200 kb, and the size of the haplotype blocks ranged from 22 bp to 199,266 bp. These haplotype blocks covered 1,620,979 bp of genetic information in the bovine genome. As depicted in Figure 1, the haplotype blocks were not evenly distributed. Instead, haplotype blocks were likely distributed according to the length of each chromosome and the density of the markers. The large number of haplotypes verified the existence of high LD in the BovineHD chip and validated the merits of haplotype analysis.

### Population stratification assessment

**Figure 2:**
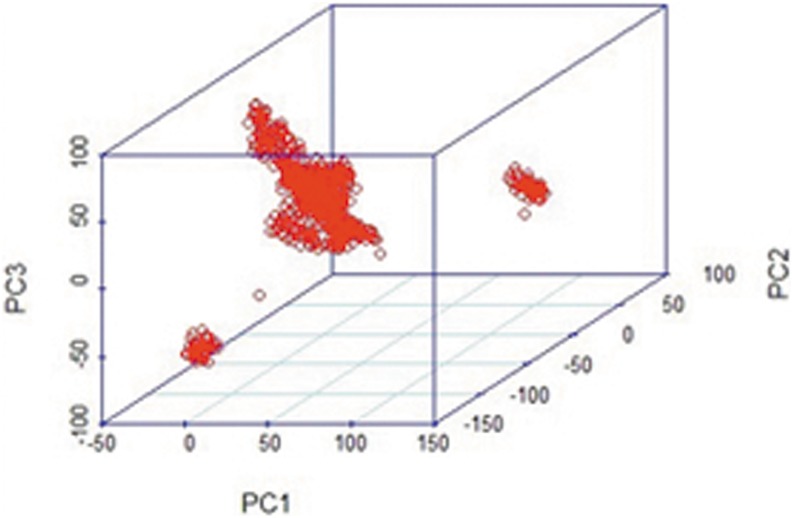
Population structure map drawn from the first three principal components. Using the R package, three principal components of markers for each individual are used to show the population stratification of the reference population.

**Figure 3:**
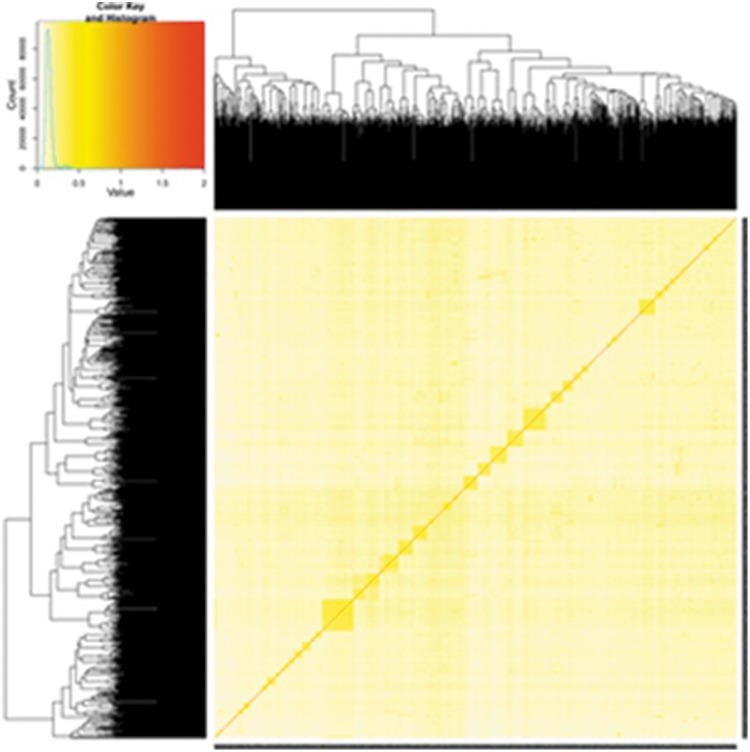
Kinship among individuals in the reference population.

Using a portion of the SNPs, we constructed and plotted the population structure based on the results of a principal component analysis (PCA). As illustrated in Figure 2, the structure of this population was drawn based on the top three eigenvectors using principal component 1 (PC1), principal component 2 (PC2), and principal component 3 (PC3). The three major distributed sectors indicate apparent population stratification in the reference samples, making a population stratification correction prerequisite in this analysis. The population stratification may have occurred because the cattle used in this study were collected from different farms and had different genetic backgrounds. In Figure 3, the kinship among individuals calculated using the classical equation from Vanden [23] is plotted, clearly illustrating the genetic relationships within the reference population.

### Significant SNPs

**Figure 4:**
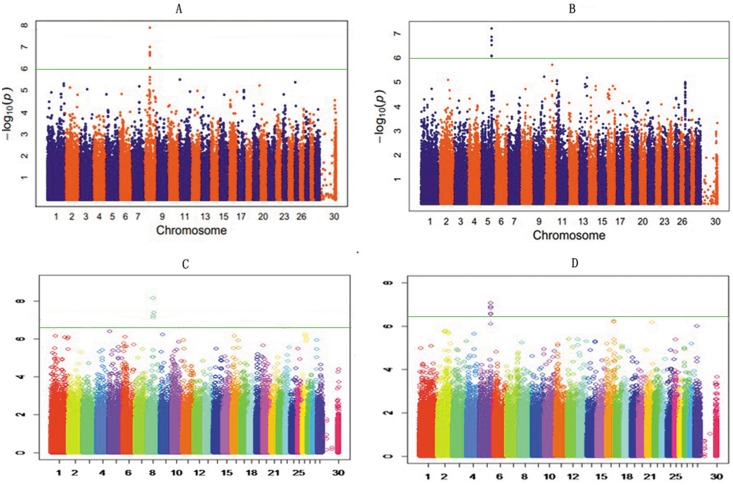
Manhattan plots of genome-wide association studies for two target traits. A and B are the plots for foreshank weight and triglyceride levels in the single marker analysis, respectively. C and D are the plots for foreshank weight and triglyceride levels in the haplotype analysis, respectively. Chromosome 1–30 is shown separated by colors, and marker positions are indicated by the ticks on the horizontal axis. In each plot, the genome-wide significance level is shown as a horizontal reference line. The single marker results for both traits show several overlapping SNPs in each map, and this apparent overlap may easily lead to misunderstandings. These SNPs are actually located within close physical proximity, and the P values are nearly the same or even identical.

**Figure 5:**
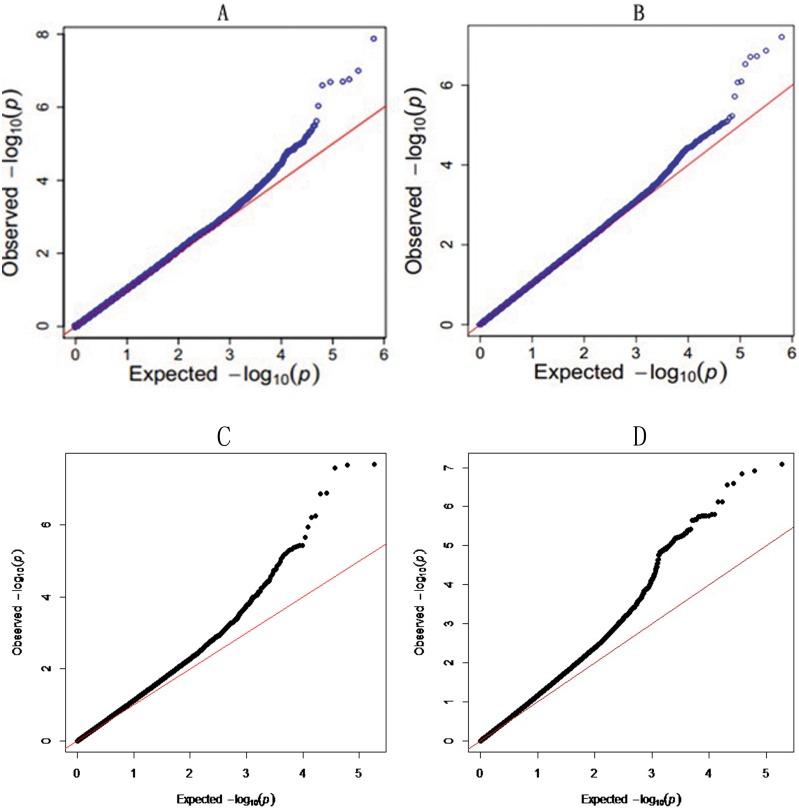
Quantile–quantile (Q–Q) plots of the genome-wide association studies. This result shows the Q–Q plots of the observed P values versus the expected P values under the null hypothesis that there was no association.

The profiles of P values (in terms of –log[p]) of all tested SNPs for the foreshank weight and the triglyceride levels are shown in Figures 4A and 4B, respectively. The details of the genomically significant SNPs detected using the single marker analysis for the two investigated traits are presented in Table 1 and Table 2, including their heritability, their positions in the genome, the nearest known genes, and the raw P values. The total number of significant SNPs identified using the single marker regression model was 12 and 7 for the corresponding two traits, foreshank weight and triglyceride levels, respectively. Specifically, for the association analysis using the weight of foreshank, all of the 12 significant SNPs were distributed closely in terms of physical distance, from 54.1 Mb to 54.5 Mb in BTA 8. These SNPs were mainly possessed by or adjacent to genes GNAQ and CEP78. Additionally, the total heritability obtained by all 12 significant markers was 58.36. For the association analysis using the triglyceride levels, 7 significant SNPs were located in close proximity to one another, from 95 Mb to 97 Mb in BTA 5, and they are found in the adjacent genes GRIN2B and ATF7IP. The heritability explained by the 7 significant SNPs was 32.96. The quantile–quantile (Q–Q) plots for the test statistics using a mixed linear model (MLM) shown in Figures 5A and 5B indicate that there is no inflation of statistics or overall systematic bias caused by the population stratification. That is, the observed test statistics generally agreed with the expected values; however, the values for the significant SNPs were above the expected values, which markedly surpassed the genome-wide significance level.

**Table 1:** Associated SNPs and nearby candidate genes for foreshank weight.

SNP	Chr	Position	P value	Heritability	Nearest gene	
					Name	Distance (bp)
BovineHD0800016290	8	54140695	1.33E-08	4.34	GNAQ	Within
BovineHD0800016379	8	54381564	1.01E-07	5.33	CEP78	52101
BovineHD0800016396	8	54443407	1.73E-07	4.48	CEP78	Within
BovineHD0800016286	8	54131549	2.00E-07	4.10	GNAQ	Within
BovineHD0800016392	8	54435508	2.06E-07	4.10	CEP78	Within
BovineHD0800016404	8	54465071	2.06E-07	4.48	CEP78	Within
BovineHD0800016406	8	54473267	2.06E-07	4.48	CEP78	4742
BovineHD0800016302	8	54167336	2.52E-07	4.82	GNAQ	Within
BovineHD0800016349	8	54281748	2.52E-07	9.08	GNAQ	1090
BovineHD0800016353	8	54291081	2.52E-07	3.97	GNAQ	10423
BovineHD0800016309	8	54183380	9.21E-07	4.59	GNAQ	Within
BovineHD0800016317	8	54204895	9.21E-07	4.59	GNAQ	Within

**Table 2:** Associated SNPs and nearby candidate genes for triglyceride levels.

SNP	Chr.	Position	P value	Heritability	Nearest gene	
					Name	Distance (bp)
BovineHD0500027280	5	96135744	6.18E-08	5.01	GRIN2B	273060
BovineHD0500027277	5	96120620	1.36E-07	4.39	ATF7IP	261657
BovineHD0500027310	5	96183375	1.88E-07	4.89	GRIN2B	225429
BovineHD0500027268	5	96080574	1.95E-07	5.14	ATF7IP	221611
BovineHD0500027313	5	96185924	2.97E-07	4.67	GRIN2B	222880
BovineHD0500027311	5	96184216	8.09E-07	4.18	GRIN2B	224588
BovineHD0500027272	5	96090773	8.53E-07	4.68	ATF7IP	231810

### Significant Haplotypes

**Figure 6:**
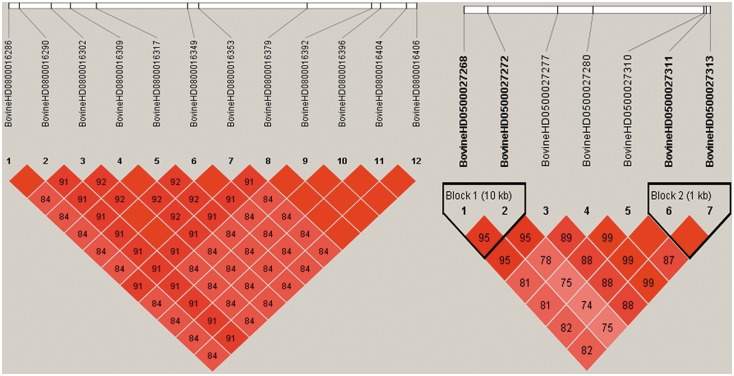
The extent of linkage disequilibrium among the 12 significant SNPs for foreshank weight and the 7 significant SNPs for triglyceride levels.

**Figure 7:**
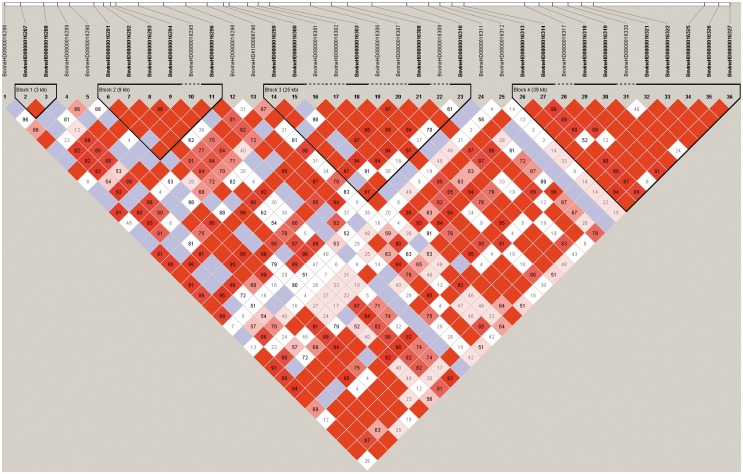
Potential haplotype structure of the hotspot area for influencing the carcass weight component. A total of 36 SNPs constitute this strong linkage disequilibrium region in chromosome 8. Solid lines mark the identified block.

In addition to the single marker analysis, Manhattan plots of the P value for each SNP against the genomic coordinates of each block were adopted to illustrate the results. Because there were no issues with overlap, the physical location of the first SNP was used to plot the graph. The P values of all tested haplotype blocks for foreshank weight and triglyceride levels are also shown in Figures 4C and 4D, respectively. The details of the genomically significant haplotype blocks detected using the haplotype-based method for the two target traits are also shown in Tables 3 and 4, including the starting and ending positions in the genome of the haplotype blocks, the number of SNPs they contain, the nearest known genes, and the raw P values. A total of 4 and 5 significant haplotype blocks were identified using a haplotype regression model for the corresponding traits of foreshank weights and triglyceride levels, respectively. Consistent with the results using the single marker analysis, all 4 significant haplotypes consistently converged on BTA 8 for the association analysis with foreshank weight. Two genes (GNAQ and CEP78) already detected in the single marker analysis and one gene (MGC134066) newly identified in the haplotype-based analysis constituted the associated gene information for foreshank weight. For the association analysis with the triglyceride levels trait, all 5 significant haplotypes were located in a similar region on BTA 5 and closely related to two genes (gene GRIN2B and ATF7IP). Most of the significant SNP regions detected using the individual SNP analysis were also identified in the haplotype-based analysis, suggesting strong LD among the detected SNPs. To test this hypothesis, we selected the significant SNPs and created the linkage map depicted in Figure 6. The majority of the SNPs were in high LD with other significant SNPs, and some pairs of significant SNPs were in complete LD. For the association analysis with foreshank weight, a region of nearly 0.1 Mb was strongly associated with the target trait and was located in the GNAQ gene, which is important in growth and development. Thus, we utilized the 36 SNPs contained in this region to infer the LD level and to estimate the combination of the superior haplotype, as shown in Figure 7. The Q–Q plots for the test statistics from the general linear model (GLM) shown in Figures 5C and 5D suggest that there is no inflation of statistics or overall systematic bias caused by population stratification in the haplotype-based analysis.

**Table 3:** Associated haplotype blocks and nearby candidate genes for foreshank weight.

NSNP	Chr	Start	End	SNP1	SNP2	P value	Cover	Nearest gene	
							Sig SNP	Name	Distance (bp)
7	8	54443407	54467908	BovineHD0800016396	BovineHD0800016405	6.69E-09	Y	CEP78	Within
7	8	59583900	59617549	BovineHD0800017790	BovineHD0800017800	4.03E-08	N	MGC134066	3252
6	8	54284149	54351569	BovineHD0800016350	Hapmap49140-BTA-38265	5.93E-08	Y	GNAQ	3491
9	8	54194680	54233883	BovineHD0800016313	BovineHD0800016327	7.52E-08	Y	GNAQ	Within

**Table 4:** Associated haplotype blocks and nearby candidate genes for triglyceride levels.

NSNP	Chr	Start	End	SNP1	SNP2	P value	Cover	Nearest gene	
							Sig SNP	Name	Distance (bp)
28	5	96135744	96177119	BovineHD0500027280	BovineHD0500027309	8.25E-08	Y	GRIN2B	273060
2	5	96183375	96184216	BovineHD0500027310	BovineHD0500027311	1.23E-07	Y	GRIN2B	225429
9	5	96069476	96093159	BovineHD0500027264	BovineHD0500027274	1.43E-07	Y	ATF7IP	210513
2	5	96185924	96186658	BovineHD0500027313	BovineHD0500027314	2.58E-07	Y	GRIN2B	222880
4	5	96112564	96128475	BovineHD0500027275	BovineHD0500027279	2.77E-07	N	ATF7IP	253601

## Discussion and Conclusions

In this study, both the individual marker analysis and the haplotype-based method identified significant associations of two quantitative traits with comparable genomic regions. However, the distributions of the P values along the genome were slightly different between the two methods. Some associations were detected using only the individual marker analysis, whereas others were found using only the haplotype-based analysis. Some inconsistencies were observed between the two methods, suggesting that the efficiency of the method may be highly dependent upon the nature of the data. Interestingly, most of the previous studies restricted their comparisons of the performance of different methods to a small subset of the genome. Our data showed that there was no apparent difference between the two methods in terms of test statistic values determined from Manhattan plots, especially for those results that were significant. However, a greater number of significant variables were detected by the single marker analysis than by the haplotype-based method. In this latter method, because of the close linkages between consecutive SNPs potentially located in the same regions, the effects were combined collectively into blocks, reducing the number of significant associations.

Recently, the detection of genes has revealed numerous genetic explanations of economic traits in beef cattle. In the present study, a carcass trait, foreshank weight, was significantly associated with 12 SNPs and 4 haplotypes located in an area of approximately 54 Mb on BTA 8. This result is consistent with those of previous studies examining the genetic associations of a carcass trait in commercial Angus cattle [24], the body weights of African cattle [25], and the average daily weight gain in Angus cattle [26]. These studies all reported significant quantitative trait loci (QTL) peaking at a similar area, 51 Mb to 55 Mb on BTA 8. The GNAQ protein reportedly participates in GTPase activity, skeletal system development, regulation of catenin importation into the nucleus, and the negative regulation of protein kinase activity. The CEP78 protein is localized in the centrosome and MGC134066 is a transmembrane protein. All of these proteins potentially regulate the growth of cattle and thus their body weights. The triglycerides examined in the present study were significantly associated with 7 SNPs and 5 haplotypes within a physical distance of approximately 96 Mb on BTA 5. Although we did not detect major regulatory fatty acid genes, such as CAST and CAPN1, in our analysis and neither method directly localized the results to a specific gene, our results were consistent with those of previous studies in terms of the levels of certain special fatty acids, for instance, the discovery of the genomic associations of trans-vaccenic acid in a Charolais × Holstein crossbred population [27], sirloin fat depth in a commercial Angus-cross population [28], and fat thickness at the twelfth rib in hybrid steers [29]. All of the above analyses mapped the associated QTL in a similar area, including the study that detected the nearby PPARA gene, which reportedly greatly influences the metabolism of fatty acids [28], [30].

To further eliminate potential associations due to population structure, we fitted the first three principal components derived from all the SNP genotypes as covariates to capture false associations attributable to population structure. This analysis sacrificed statistical power for mutations with effects confounded by population structure. In previous studies that did not incorporate the population structure into the analysis, the P value was often overestimated and the Q–Q plots markedly deviated from the expectation. For a haplotype-based analysis, it is unrealistic to incorporate the kinship matrix using the MLM because the complex indicator variables may decrease the accuracy of equation and even lead to a result that deviates from the original definition of kinship. In addition, the large sample size would create a huge computational burden for the mixed model analysis because the computing time increases cubically with a large number of individuals. In terms of the MLM, some studies [31] assert that the statistical model proposed by Zhang [32] has higher statistical power than approaches that consider population structure only or those that consider both population structure and individual relationships without grouping. Our results verify this assertion and find that the statistical power is closely related to the definition of the groups.

Although QTL mapping for economically important traits in domestic animals has already achieved remarkable results, only a small percent of the genetic variation has been captured because of the low density of the markers. GWAS, which utilize high-density SNPs, provide a new way to confront this issue. With the imputation strategy, using lower density markers may offer a few advantages with respect to lower costs. However, that strategy would introduce an additional imputation error rate in the already high false discovery rates that exist for association studies. In current GWAS, how to best reduce the data dimension is a thorny problem. When markers are divided into many blocks based on their LD levels, a block or group of markers is considered as one super marker. Only effects of the marker groups are estimated and tested, efficiently avoiding the little knowledge about the interactions between markers. This solution substantially reduces the data dimension and makes it possible to detect the interactions (epispastic effects) at the gene level. Hu et al [33] established a new method for analyzing large datasets, developing a bin model to predict genomic values based on an infinitesimal model. Nevertheless, haplotype blocks are natural choices as grouping markers in the bin model compared with the rigid definition of evenly distributed blocks. Theoretically, multiple traits can be analyzed simultaneously in a single model; however, traits were examined separately in the present study due to the lack of an accurate model. Many studies have proven the superiority of multiple trait association studies over association studies using individual traits [34]–[36]. Even so, there is no consensus on a consistent model that might further improve multiple trait GWAS [37].

In summary, this is the first broad application of the GWAS method using single marker-based and haplotype-based analyses, as well as haplotype construction, in Simmental cattle. We partitioned the whole cattle genome into 92,553 haplotype blocks using the standard expectation–maximum (EM) algorithm. The results showed that the regions of high LD extended up to approximately 200 kb and that the size of haplotype blocks ranged from 22 bp to 199,266 kb. The GWAS found 12 and 7 SNPs significantly genomically associated with foreshank weight and triglyceride levels, respectively. By contrast, the haplotype-based association found that 4 and 5 haplotype blocks contained the majority of the significant SNPs that were strongly associated with foreshank weight and triglyceride levels, respectively. In addition, 2 and 3 genes, respectively, were detected in the significant region and might be responsible for the variation of the two traits.

In conclusion, this study provides important information on the genetic mechanisms of two traits in beef cattle and on the structure of the genome in Simmental cattle.

## Materials and Methods

### Ethics statement

All animal procedures were conducted with strict adherence to the guidelines proposed by the Chinese Council on Animal care, and all protocols were approved by the Science Research Department of the Institute of Animal Science at the Chinese Academy of Agricultural Sciences (Beijing, China). Samples were collected during regular quarantine inspections on the farms. The farm owners approved the use of the animals and private land for this study.

### Animal resources and phenotypes

The training population consisted of 942 young Simmental bulls born in 2008–2011 and is part of a resource population established in Inner Mongolia. Each individual was observed and measured for growth and developmental traits until they were slaughtered when they were 16 to 18 months old. During slaughter, carcass traits and meat quality traits were measured according to the Institutional Meat Purchase Specifications for fresh beef guidelines. The two target traits were measured as follows. After removing the exposed fat and tendons, the weight of foreshank was determined for the high quality meat, which was kept fresh and intact, from the elbow to the wrist while maintaining strict compliance with the rules stated in GB/T 27643-2011. For triglyceride levels, samples were taken in accordance with GB/T 2223-2008 from the loin eye muscle and extracted according to the procedure described by Sukhija (1998). Subsequently, the triglyceride samples were analyzed using gas chromatography (gas chromatograph, GC-2014 CAFsc, Shimadzu Scientific Instruments) under the following conditions: the temperature of the inlet was 220°C. The oven was heated up to 250°C at a rate of 5°C per min and then maintained at 220°C. Each analysis took 20 min. After collecting the original data, fixed effects, including years, farm, and month of birth, were corrected in advance using the following equation:
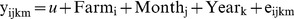
(1)where ***y_ijkm_*** is the vector of phenotype, ***u*** is the population mean, **Farm_i_** is the category of the farm where the animal was raised, **Month_j_** is the number of months after birth, **Year_k_** is the year of slaughter, ***e_ijkm_*** is the random residual. The residual ***e_ijkm_*** was subsequently used for the study examining the genomic associations with SNPs and haplotypes.

### Genotyping and quality control

Semen or blood samples were collected during the regular quarantine inspection of the farms. Genomic DNA was extracted from blood samples using a TIANamp Blood DNA Kit (Tiangen Biotech Company limited, Beijing, Chain), and DNA with an A260/280 ratio between 1.8 and 2.0 were subjected to further analysis. All individuals were genotyped using the Illumina BovineSNP BeadChip containing 774,660 SNPs. The mean value of the distance between each marker was 3.43 kb, and the variance value of the distance between each marker was 19.19 Mb. The genotyping platform adopted in this study was Illumina’s Infinium II Assay (Illumina Inc., San Diego, CA, USA). Samples were genotyped using Illumina’s BeadStudio data analysis software, and SNP chips were scanned using iScan and analyzed using Infinium GenomeStudio (Illumina Inc.).

Prior to statistical analysis, the SNP data were pre-processed and the following SNPS were removed: SNPs with call rates less than 90%, minor allele frequencies less than 5%, genotype appearances in less than five individuals, and marked departure from the Hardy-Weinberg equilibrium (with lower than 10^−6^ probability). Moreover, individuals with more than 10% missing genotypes or those above a 2% Mendelian error rate for SNP genotypes were excluded. Thus, a total of 942 individuals and 631,396 SNPs remained in the sample for the subsequent analysis.

### Haplotype block partitioning algorithms

The history of recombination between a pair of SNPs can be estimated with the use of the normalized measure of allelic association, D’ [38]. Using the definition given by Gabriel [39], the pairs were considered in “strong LD” if the one-sided upper 95% confidence boundary of D’ was >0.98 (that is, consistent with no historical recombination) and the lower boundary was above 0.7 [39]. If the D’ for a pair of SNPs was lower than 0.7, then the next haplotype block began. That is to say, only nearby SNPs with continuous combinations were included in a haplotype block, and few SNPs having low LD with adjacent markers were omitted in the haplotype-based association study. After the detection of haplotype blocks, haplotypes and their frequencies for an individual were obtained with the standard expectation**–**maximum (EM) [40] algorithm. Specifically, the EM iteration alternates between performing an expectation (E) step, which creates a function for the expectation of the log-likelihood evaluated using the current estimate for the haplotype and its frequency, and a maximization (M) step, which computes haplotype and its frequency maximizing the expected log-likelihood found on the E step. These parameter-estimates are then used to determine the distribution of the latent variables in the next E step. The reference threshold of SNPs within a close linkage haplotype and the partitioning standard can be founded in the user manual of the PLINK software [41].

### Single-marker association studies

Association data for each SNP via regression analysis were presented based on the MLM below:
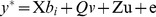
(2)where y* is the adjusted phenotype, **b_i_** is the regression coefficient of phenotype on SNP genotypes, **X** is the vector of the SNP genotype indicators and takes values 0, 1, or 2 corresponding to the three genotypes 11, 12 and 22, **v** is the effect of population structure, **Q** is the corresponding principal components matrix, **u** is the vector of the residual polygenetic effects with **u∼N(0, K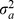)** (where **K** is the genetic relationship calculated by the markers, 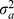 is the additive variance), **Z** is the corresponding matrix, and **e** is the vector of residual errors with **e∼N(0, I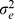)**. The population structure was quantified using the first three eigenvectors as covariates in this model. A *t*-test statistic was used to determine the significance of each SNP and was calculated as follows:
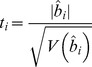
(3)where 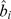 is the estimate of b and the corresponding variance V(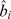) can be obtained via mixed model equations (MME). For both analyses, the Bonferroni method was adopted to adjust for multiple testing from the number of SNP loci detected. A SNP was considered significant at the genome level if the raw P value was less than 0.05/N, where N is the number of SNP loci tested in analyses. However, with 631,396 markers, the Bonferroni correction was too stringent for the potential associated SNPs. Thus, in the single marker analyses, the significance threshold for the P value was set at 1.0×10^−6^. The significance threshold for the P value of the haplotype was set based on the equation at 5.4×10^−7^ (0.05/92,553).

### Haplotype-based association studies

Different from the single marker analysis, a GLM was adopted to perform the regression analysis in the haplotype-based association studies as shown below:
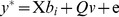
(4)where y* is the adjusted phenotype, **b_i_** is the regression coefficient of phenotype on haplotype block, **X** is the vector of the corresponding haplotype indicators, **v** is the effect of population structure, **Q** is the corresponding principal components matrix and **e** is the vector of residual errors with **e∼N(0, I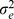)**. Based on equation (4), the *t*-test statistic was also used for the significance test. An MLM was not adopted here mainly because of the complicated process for attaining the genetic relationship constructed by the haplotypes. Moreover, rarely seen haplotypes and individuals with low frequency were eliminated before the association study to avoid an adverse impact on the significance test.
